# Correlation between optic nerve head circulation and visual function before and after anti-VEGF therapy for central retinal vein occlusion: prospective, interventional case series

**DOI:** 10.1186/s12886-016-0211-7

**Published:** 2016-04-05

**Authors:** Daisuke Nagasato, Yoshinori Mitamura, Kentaro Semba, Kei Akaiwa, Toshihiko Nagasawa, Yuki Yoshizumi, Hitoshi Tabuchi, Yoshiaki Kiuchi

**Affiliations:** Department of Ophthalmology, Saneikai Tsukazaki Hospital, Himeji, Japan; Department of Ophthalmology and Visual Sciences, Graduate School of Biomedical Sciences, Hiroshima University, Hiroshima, Japan; Department of Ophthalmology, Institute of Biomedical Sciences, Tokushima University Graduate School, 3-18-15 Kuramoto, Tokushima, 770-8503 Japan

**Keywords:** Anti-vascular endothelial growth factor agent, Central retinal vein occlusion, Fundus-related microperimetry, Laser speckle flowgraphy, Retinal blood flow

## Abstract

**Background:**

To determine the correlation between the optic nerve head (ONH) circulation determined by laser speckle flowgraphy and the best-corrected visual acuity or retinal sensitivity before and after intravitreal bevacizumab or ranibizumab for central retinal vein occlusion.

**Methods:**

Thirty-one eyes of 31 patients were treated with intravitreal bevacizumab or ranibizumab for macular edema due to a central retinal vein occlusion. The blood flow in the large vessels on the ONH, the best-corrected visual acuity, and retinal sensitivity were measured at the baseline, and at 1, 3, and 6 months after treatment. The arteriovenous passage time on fluorescein angiography was determined. The venous tortuosity index was calculated on color fundus photograph by dividing the length of the tortuous retinal vein by the chord length of the same segment. The blood flow was represented by the mean blur rate (MBR) determined by laser speckle flowgraphy. To exclude the influence of systemic circulation and blood flow in the ONH tissue, the corrected MBR was calculated as MBR of ONH vessel area – MBR of ONH tissue area in the affected eye divided by the vascular MBR – tissue MBR in the unaffected eye. Pearson’s correlation tests were used to determine the significance of correlations between the MBR and the best-corrected visual acuity, retinal sensitivity, arteriovenous passage time, or venous tortuosity index.

**Results:**

At the baseline, the corrected MBR was significantly correlated with the arteriovenous passage time and venous tortuosity index (*r* = -0.807, *P* < 0.001; *r* = -0.716, *P* < 0.001; respectively). The corrected MBR was significantly correlated with the best-corrected visual acuity and retinal sensitivity at the baseline, and at 1, 3, and 6 months (all *P* < 0.050). The corrected MBR at the baseline was significantly correlated with the best-corrected visual acuity at 6 months (*r* = -0.651, *P* < 0.001) and retinal sensitivity at 6 months (*r* = 0.485, *P* = 0.005).

**Conclusions:**

The pre-treatment blood flow velocity of ONH can be used as a predictive factor for the best-corrected visual acuity and retinal sensitivity after anti-VEGF therapy for central retinal vein occlusion.

**Trial registration:**

Trial Registration number: UMIN000009072. Date of registration: 10/15/2012.

**Electronic supplementary material:**

The online version of this article (doi:10.1186/s12886-016-0211-7) contains supplementary material, which is available to authorized users.

## Background

A central retinal vein occlusion (CRVO) is one of the major causes of vision reduction, and anti-vascular endothelial growth factor (VEGF) agents have been shown to significantly improve visual acuity in eyes with macular edema (ME) due to a CRVO [1].

Laser speckle flowgraphy (LSFG) is a non-invasive method of real-time measurements of the blood flow on the optic nerve head (ONH), retina, and choroid [2–6]. It can measure the relative blood flow velocity, called the mean blur rate (MBR), that has been shown to be significantly correlated with the actual blood flow rate determined by the hydrogen gas clearance method and the microspheres technique [7, 8]. It has been reported that LSFG can record the blood flow from the same location of an eye with high reproducibility especially on the ONH [9]. This then provides an accurate way to monitor the circulation changes before and after pharmacological interventions [10].

Yamada et al. reported that the MBR values in large ONH vessels measured by LSFG were correlated with the higher aqueous VEGF concentrations in eyes with CRVO [11]. Nagaoka et al. reported that a single intravitreal bevacizumab (IVB) injection did not affected the retinal microcirculation in eyes with acute branch retinal vein occlusion (BRVO) for at least 3 months after the injection [12]. However, there has been no report that compared the ocular circulation before and after anti-VEGF therapy for CRVO except for a report of 3 cases in which the statistical association between ONH circulation and visual function was not determined [13].

Thus, the purpose of this study was to examine the relationship between the retinal blood flow and visual acuity or retinal sensitivity in 31 eyes before and after IVB or intravitreal ranibizumab (IVR) for CRVO.

## Methods

This was a prospective, interventional case series of 31 eyes of 31 treatment-naïve patients (20 men and 11 women) with unilateral CRVO. The patients were examined by LSFG and microperimetry at the baseline and at 1, 3, and 6 months after IVB or IVR for ME due to a CRVO. The fluorescein arteriovenous passage time was determined at the baseline. All patients were examined within 3 month of the onset of the symptoms. The age of the patients at presentation ranged from 40 to 83 years (mean, 66.9 years). All treatment-naïve patients who were diagnosed with ME due to CRVO, had a healthy fellow eye, and had IVB or IVR injections in the Department of Ophthalmology of Saneikai Tsukazaki Hospital from October 2012 through February 2015 were studied. Approval was obtained from the Institutional Review Board of Saneikai Tsukazaki Hospital prior to beginning this study, and the patients gave their written informed consent prior to their inclusion. The patients have provided permission to publish clinical data of their case in this study. This study was registered with the University hospital Medical Information Network (UMIN) clinical trials registry. The registration title is “UMIN000009072, Correlation between optic nerve head circulation and retinal sensitivity before and after anti-VEGF drug intravitreal injection for central retinal vein occlusion” (October 15, 2012). The procedures used in this study adhered to the tenets of the Declaration of Helsinki.

Patients with ME due to a CRVO, central foveal thickness of ≥250 μm in the optical coherence tomographic (OCT) images, and a decimal visual acuity from 0.01 to 0.8 were studied. Patients with a history of cerebral infarction, anti-VEGF therapy, vitrectomy, and uveitis, or other vitreoretinal diseases were excluded. In addition, patients with uncontrolled high blood pressure, diabetes, intraocular pressure (IOP) of 21 mmHg or more, or iris neovascularization were excluded.

The blood flow of the major blood vessels on the ONH was measured in all patients, and the arteriovenous passage time of the retina was determined by fluorescein angiography (FA). A previous study investigating CRVO with FA [14] showed that more than 10 disc areas of the nonperfusion areas were observed in the ischemic type of CRVO. Thus, a diagnosis of ischemic CRVO was made in eyes with more than 10 disc areas of the nonperfusion areas [11]. The venous tortuosity index was calculated on color fundus photograph.

Patients who started their treatment between October 2012 and August 2013 received IVB injections (1.25 mg/0.05 mL), and those who started treatment between September 2013 and February 2015 received IVR injections (0.5 mg/0.05 mL). After the initial treatment, the patients were examined once a month. When the central foveal thickness was ≥250 μm, or the physician determined that additional treatment was necessary, the patients received additional injections of the same anti-VEGF agent.

All patients had a standard ophthalmologic examination including IOP measurements before and after IVB or IVR. The best-corrected visual acuity (BCVA) was measured with a standard Japanese Landolt visual acuity chart, and the decimal visual acuity was converted to the logarithm of the minimal angle of resolution (logMAR) units for statistical analyses. The anterior and posterior segments were examined by slit-lamp biomicroscopy, indirect ophthalmoscopy, color fundus photography, and spectral-domain OCT (SD-OCT). The systolic blood pressure (SBP), diastolic blood pressure (DBP), and heart rate were measured. The mean arterial pressure (MAP) and the mean ocular perfusion pressure (MOPP) were calculated according to the following formulas and used for the analyses: MAP = DBP + 1/3 (SBP - DBP), and MOPP = 2/3 MAP – IOP.

### Laser speckle flowgraphy

**Fig. 1:**
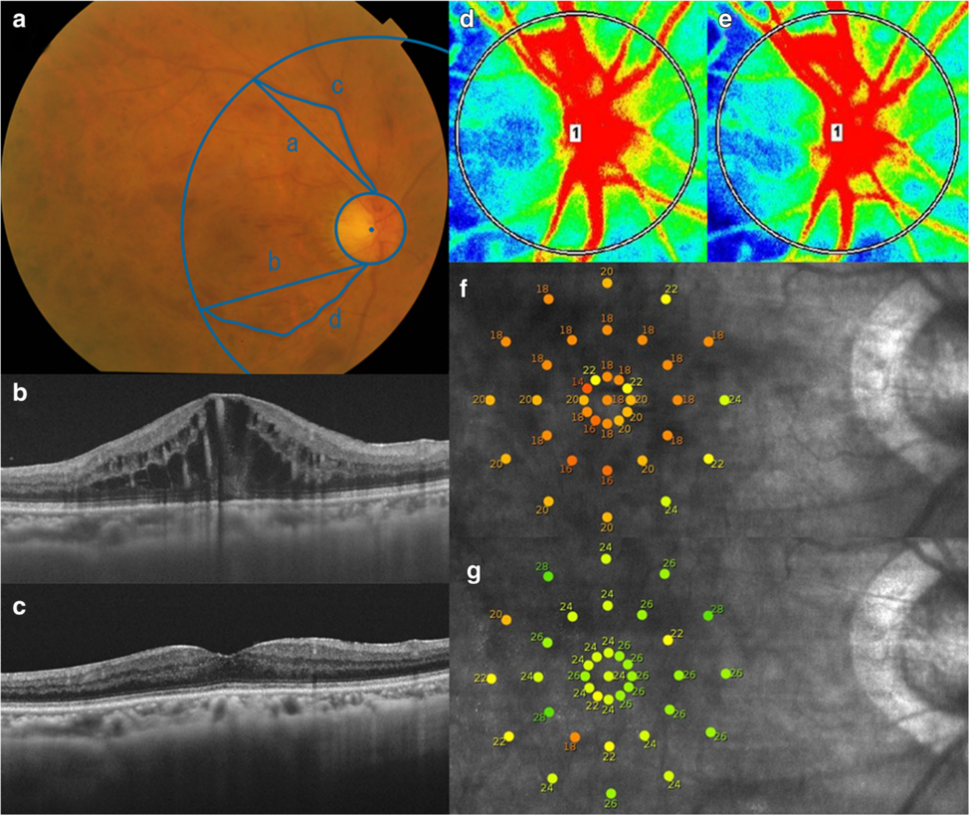
Ophthalmologic examination images before and 6 months after anti-VEGF therapy for central retinal vein occlusion. Fundus photograph, spectral-domain optical coherence tomographic (SD-OCT) images, laser speckle flowgraphic (LSFG) images, and microperimetric maps before and 6 months after an initial anti-VEGF therapy of the right eye of a 66-year-old woman with cystoid macular edema (CME) due to a central retinal vein occlusion are presented. The decimal best-corrected visual acuity was 0.5 before the treatment and 1.0 at 6 months after the treatment. **a**: Fundus photograph at the baseline. The venous tortuosity index was calculated on color fundus photograph. Measurements of superior and inferior venous arcades were obtained starting from the optic disc margin to the crossing point of a circle whose diameter is the distance from the center of optic disc to the fovea. The course of the veins was traced using Photoshop (Adobe Systems, Inc. Ca, USA). NIH ImageJ software was used to measure the lengths of the tortuous vein (c and d) and chord of the vessels (a and b). The venous tortuosity was calculated by dividing the length of the tortuous retinal vein by the chord length of the same segment (c/a and d/b). The average of the venous tortuosity ((c/a + d/b)/2) was calculated to obtain the venous tortuosity index. **b**: SD-OCT image at the baseline showing CME. **c**: SD-OCT image at 6 months showing a resolution of the CME. **d**: A false-color composite map of the optic nerve head was created using the LSFG findings at the baseline. The red area indicates a faster blood flow, and the blue area indicates a slower blood flow. **e**: A false-color composite map by LSFG at 6 months. There is no obvious difference in the blood flow as compared with the LSFG map at the baseline (**d**). **f**: Microperimetric map image at the baseline. A total of 37 stimulus locations covering the central 10° field were tested. The mean retinal sensitivity at the 37 locations is 19.1 dB. **g**: Microperimetric map image at 6 months. The mean retinal sensitivity at the 37 locations is 24.5 dB

**Fig. 2:**
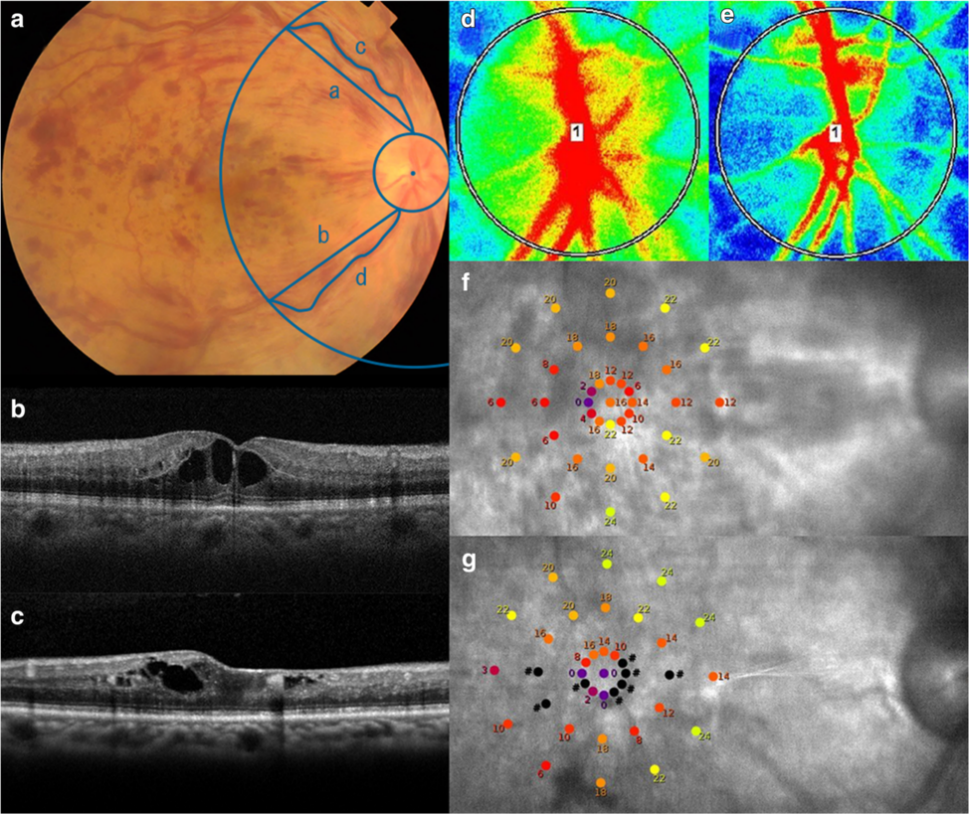
Ophthalmologic examination images before and 6 months after anti-VEGF therapy for central retinal vein occlusion. Fundus photograph, spectral-domain optical coherence tomographic (SD-OCT) images, laser speckle flowgraphic (LSFG) images, and microperimetric maps before and 6 months after an initial anti-VEGF therapy of the right eye of a 66-year-old man with cystoid macular edema (CME) due to a central retinal vein occlusion are presented. The decimal best-corrected visual acuity was 0.3 before the treatment and 0.1 at 6 months after the treatment. **a**: Fundus photograph at the baseline. Note that the venous tortuosity is larger as compared with fundus photograph presented in Fig. 1a. **b**: SD-OCT image at the baseline shows CME. **c**: SD-OCT image at 6 months indicates residual CME and retinal thinning around the fovea. **d**: A false-color composite map at the optic nerve head was created using LSFG at the baseline. The red area indicates a faster blood flow, and the blue area indicates a slower blood flow. **e**: A false-color composite map by LSFG at 6 months. Note the decrease of blood flow as compared with the LSFG map before the treatment (**d**). **f**: Microperimetric map at the baseline. The mean retinal sensitivity at the 37 locations is 14.4 dB. **g**: Microperimetric map image at 6 months. The mean retinal sensitivity at the 37 locations is 10.6 dB

**Fig. 3:**
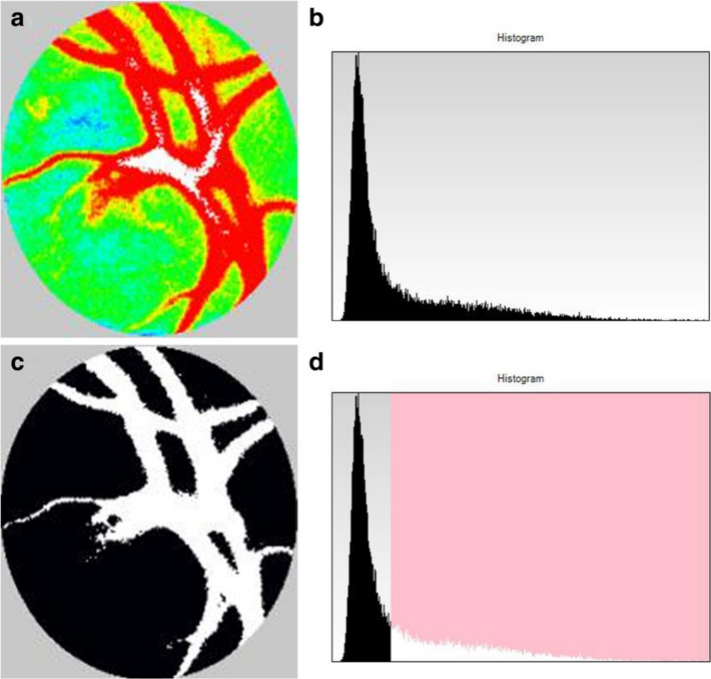
Composite maps and histograms at the optic nerve head (ONH) created by laser speckle flowgraphy. **a**: A false-color composite map at the ONH. **b**: A histogramic analysis of the ONH. The vertical axis represents the number of pixels and the horizontal axis represents the mean blur rate. **c**: A binary format image for segmentation between the vessel (white area) and tissue (black area) areas. **d**: A histogram analyzed using the built-in image viewer software that uses an automated definitive threshold. The area to the left of the threshold line corresponds to the ONH tissue (black area in **c**), whereas the area to the right of the line corresponds to the ONH vessel (white area in **c**)

LSFG-NAVI (Softcare, Fukuoka, Japan) was used to evaluate the ONH circulation in eyes with a CRVO (Figs. 1 and 2). The principles of LSFG have been reported in detail [15]. 　In brief, LSFG uses a diode laser (wavelength 830 nm) to detect the movement of the red blood cells in the blood vessels. The light scattered by the movement of the blood cells creates speckle patterns on the area where the sensor is focused, and the scattered light produces a blur in the speckle patterns. The mean blur rate, which is the change in the blur, is a quantitative value that represents the relative blood flow velocity [15–19]. The patient's pupil was dilated with Mydrin P (1 % tropicamide and 2.5 % phenylephrine) 30 min prior to the LSFG examinations. During the examinations, the patients were instructed to fixate steadily on a target light while the speckle pattern on the ONH was recorded. With an auto-tracking system, the same site can be measured for several seconds. The relative blood flow velocity is represented by the MBR and displayed as a two-dimensional color map. The MBR of the vessel area is expressed as the MV and the MBR of the tissue area as the MT. The appropriate threshold between the vessel and tissue areas is automatically determined by the built-in software, and the MBR of the area of the major arteries and veins as MV and the MBR of the tissue area as MT can be calculated (Fig. 3) [9].

When measuring the retinal circulation with LSFG, the measurements are influenced by the deep choroidal circulation. Similarly, the ONH measurements are influenced by the blood flow of the ONH tissue (MT) [15, 16]. To evaluate the major arteriovenous circulation of the ONH excluding the blood flow of the ONH tissue, the value was obtained by subtracting the MT from the MV [11]. In addition, to exclude the influence of each patient's systemic circulation at the time of the measurements and to allow for inter-patient comparisons of the measurements, a corrected MBR value was obtained by the MBR (MV minus MT) of the affected eye divided by the MBR (MV minus MT) of the fellow eye [11]. The LSFG was measured at the baseline and at 1, 3, and 6 months after the initial treatment.

### Microperimetry

The retinal sensitivity was determined by macular integrity assessment (MAIA) microperimetry (CenterVue, Padova, Italy; Figs. 1 and 2). MAIA testing was conducted in a dark room without pupil dilation. The testing conditions were similar to that described in detail in previous studies [17, 18]. Briefly, 37 loci in the 10° central macula was assessed using a 4 to 2 threshold strategy, a fixation target that consisted of a red circle with a 1° diameter, stimulus size of Goldmann III, background luminance of 4 apostilb (asb), maximum luminance of 1000 asb, and a stimulus dynamic range of 36 dB.

### Calculation of venous tortuosity index

The venous tortuosity index was calculated as previously reported (Figs. 1 and 2) [20, 21]. Briefly, color fundus photographs were obtained with a Topcon fundus camera (TRC-50DX, Topcon, Tokyo, Japan). The course of the veins was traced using the Photoshop software (Adobe Systems, Inc. Ca, USA). Measurements of the superior and inferior venous arcades were obtained starting from the ONH margin to the crossing point of a circle whose diameter was the distance from the center of ONH to the fovea. NIH ImageJ software was used to measure the length of the tortuous vein and length of the chord of the vessel. The venous tortuosity was calculated by dividing the arc length of the retinal vein by the chord length of the same segment. The average of the venous tortuosity of the superior and inferior venous arcades was calculated to obtain the venous tortuosity index.

### Statistical analyses

Repeated-measures analysis of variance (ANOVA) with Greenhouse-Geisser corrections was used to determine the significance of the changes in the BCVA, retinal sensitivity, corrected MBR, MV, MT, SBP, DBP, heart rate, IOP, MAP, and MOPP. The Bonferroni test was used for post hoc analysis. Pearson’s correlation tests were used to determine the significance of correlations between the corrected MBR and the BCVA, retinal sensitivity, arteriovenous passage time, or venous tortuosity index. A *P* value of <0.05 was considered statistically significant.

## Results

**Fig. 4:**
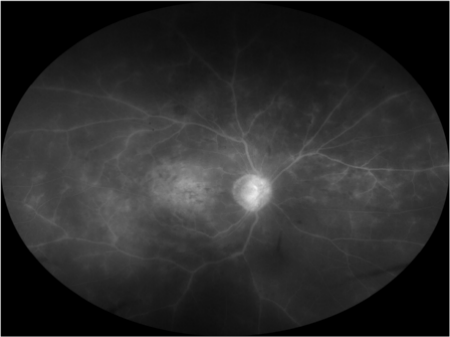
Fluorescein angiography before anti-VEGF therapy for central retinal vein occlusion (CRVO). The same case presented in Fig. 1. Because there are not more than 10 disc areas of the nonperfusion areas, this case was diagnosed with nonischemic CRVO

**Fig. 5:**
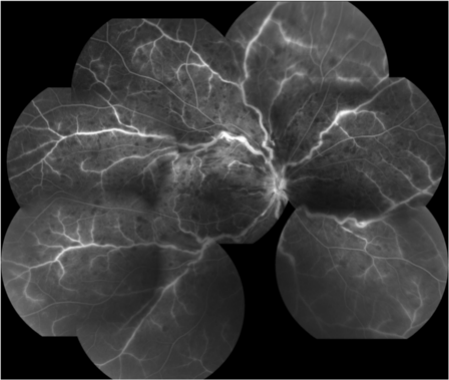
Fluorescein angiography before anti-VEGF therapy for central retinal vein occlusion (CRVO). The same case presented in Fig. 2. Because there are not more than 10 disc areas of the nonperfusion areas, this case was also diagnosed with nonischemic CRVO

All patients were followed for at least 6 months after the initial injection of IVB or IVR. IVB was performed on 15 eyes and IVR on 16 eyes. The injections were administered two to five times (3.52 ± 0.88 (mean ± SD)) to each patient during the 6 months. According to the FA findings, the diagnosis of nonischemic CRVO was made in 26 eyes (Figs. 4 and 5), and the diagnosis of ischemic CRVO was made in 5 eyes. The SBP, DBP, heart rate, IOP, MAP, and MOPP were not significantly different among the baseline and 1, 3, and 6 months after the treatments (*P* = 0.930, *P* = 0.958, *P* = 0.966, *P* = 0.745, *P* = 0.969, *P* = 0.886, respectively).

**Fig. 6:**
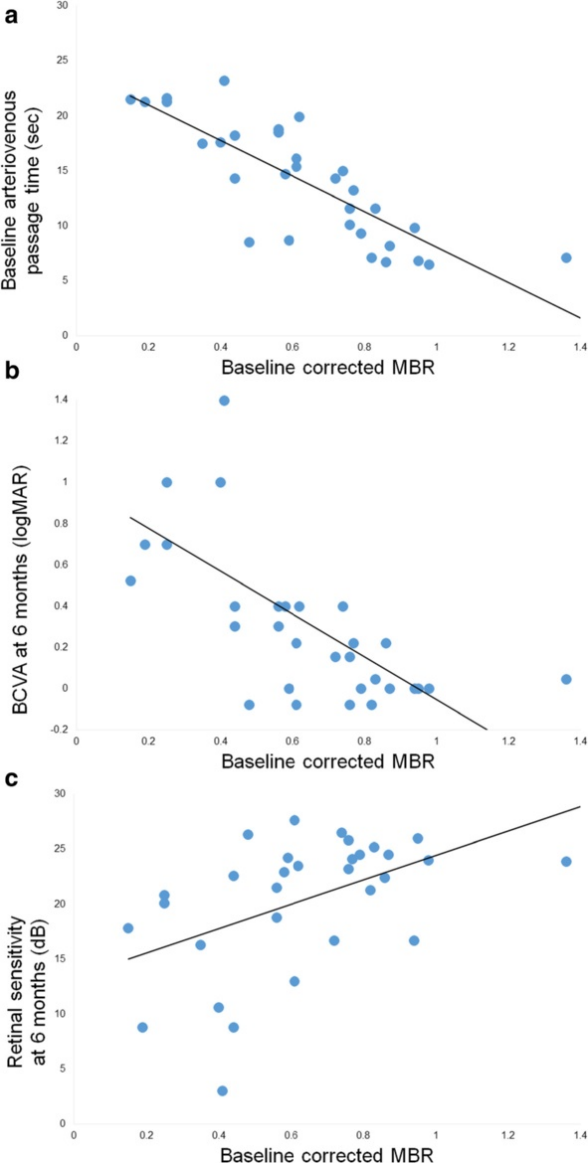
Correlation between optic nerve head (ONH) circulation and arteriovenous passage time or visual function. Correlation between the baseline mean blur rate (MBR) at the ONH and the baseline arteriovenous passage time, the best-corrected visual acuity (BCVA) at 6 months, or mean retinal sensitivity at 6 months is presented. To exclude the influence of systemic circulation and blood flow of ONH tissue, the corrected MBR was calculated as; (MBR of ONH vessel area – MBR of ONH tissue area) in the affected eye ÷ (vascular MBR – tissue MBR) in the healthy fellow eye [11]. **a**: Correlation between the baseline corrected MBR at the ONH and the arteriovenous passage time at the baseline. The corrected MBR is significantly correlated with the arteriovenous passage time on fluorescein angiography (*r* = -0.807*, P* <0.001). The solid line represents the linear regression line (y = -16.102x + 24.200). **b**: Correlation between the baseline corrected MBR at the ONH and BCVA in logMAR units at 6 months after the initial treatment. The corrected MBR is significantly correlated with the BCVA at 6 months (*r* = -0.651, *P* <0.001). The solid line represents the linear regression line (y = -1.036x + 0.983). **c**: Correlation between the baseline corrected MBR at the ONH and mean retinal sensitivity at 6 months. The corrected MBR is significantly correlated with the retinal sensitivity at 6 months (*r* = 0.485, *P* = 0.005). The solid line represents the linear regression line (y = 11.059x + 13.378)

At the baseline, the corrected MBR was 0.63 ± 0.26. The arteriovenous passage time on FA was 14.0 ± 5.2 s, and the venous tortuosity index on color fundus photograph was 1.113 ± 0.053. The corrected MBR was significantly correlated with the arteriovenous passage time (*r* = -0.807, *P* <0.001; Fig. 6a) and venous tortuosity index (*r* = -0.716, *P* <0.001). The corrected MBR after the first intravitreal injection was 0.60 ± 0.23 at 1 month, 0.61 ± 0.23 at 3 months, and 0.62 ± 0.28 at 6 months (Additional file 1). No significant differences were observed in the corrected MBR among the baseline and post-treatment times (*P* = 0.379). The MV in the affected eye was 34.8 ± 11.2 at the baseline, 32.0 ± 10.3 at 1 month, 31.6 ± 10.9 at 3 months, and 32.9 ± 12.2 at 6 months. No significant differences were observed in the MV among the baseline and post-treatment times (*P* = 0.116). The MT was 14.3 ± 4.6 at the baseline, 12.4 ± 3.8 at 1 month, 11.7 ± 4.0 at 3 months, and 12.3 ± 4.2 at 6 months. Significant differences were observed in the MT among the baseline and post-treatment times (*P* = 0.003). The MT at 1 and 3 months was significantly smaller than that at the baseline (*P* = 0.011, *P* = 0.002, respectively).

The mean BCVA was 0.68 ± 0.48 logMAR units at the baseline, 0.39 ± 0.36 logMAR units at 1 month after the first intravitreal injection, 0.36 ± 0.35 logMAR units at 3 months, and 0.33 ± 0.42 logMAR units at 6 months. Significant differences were observed in the BCVA among the baseline and post-treatment times (*P* <0.001). The BCVA at 1, 3, and 6 months was significantly better than that at the baseline (*P* <0.001, *P* = 0.001, *P* = 0.007, respectively).

The retinal sensitivity was 15.2 ± 7.5 dB at the baseline, 18.7 ± 6.6 dB at 1 month, 20.9 ± 6.0 dB at 3 months, and 20.4 ± 6.0 dB at 6 months. Significant differences were observed in the retinal sensitivity among the baseline and post-treatment times (*P* <0.001). The retinal sensitivity at 1, 3, and 6 months was significantly better than that at the baseline (*P* = 0.001, *P* <0.001, *P* = 0.001, respectively).

The MBR was significantly correlated with the BCVA (all *P* <0.005) and the retinal sensitivity (all *P* <0.050, Table 1) at all time points.

**Table 1:** Correlations between MBR on ONH and BCVA or retinal sensitivity before and after anti-VEGF therapy

	Corrected MBR on ONH			
	Baseline	1 month	3 months	6 months
BCVA (logMAR)	*r* = -0.543	*r* = -0.672	*r* = -0.546	*r* = -0.565
	*P* = 0.001	*P* < 0.001	*P* = 0.001	*P* <0.001
Retinal sensitivity (dB)	*r* = 0.572	*r* = 0.608	*r* = 0.552	*r* = 0.430
	*P* <0.001	*P* <0.001	*P* = 0.001	*P* = 0.015

BCVA, best corrected visual acuity; Corrected MBR, (MBR of ONH vessel area – MBR of ONH tissue area) in the affected eye ÷ (vascular MBR – tissue MBR) in the unaffected eye; logMAR, logarithm of the minimum angle of resolution; MBR, mean blur rate; ONH, optic nerve head; VEGF, vascular endothelial growth factor

There was a significant correlation between the corrected MBR at the baseline and the BCVA at 6 months (*r* = -0.651, *P* <0.001; Fig. 6b). There was also a significant correlation between the corrected MBR at the baseline and the retinal sensitivity at 6 months (*r* = 0.485, *P* = 0.005; Fig. 6c).

## Discussion

The results showed that the corrected MBR at the baseline was significantly correlated with the BCVA and retinal sensitivity at 6 months after anti-VEGF therapy. This indicates that the visual prognosis is good in patients with CRVO who have good blood flow on the ONH at the baseline. In a report on 3 cases with CRVO, Matsumoto et al. also suggested that the prognosis of CRVO may be predicted by measuring the MBR using LSFG [13]. In eyes with ME due to a BRVO, Nagaoka et al. reported that the visual prognosis was good when the retinal blood flow measured by laser Doppler velocimetry before IVB was good [12]. Taken together, these results suggest that the status of the blood flow prior to the treatment is a useful predictor of the post-treatment BCVA for both CRVO and BRVO.

The measurements of the LSFG had excellent reproducibility which then permitted a non-invasive method to measure the ocular circulation. Aizawa et al. used LSFG and reported that the intra-session reproducibility of the MBR in the ONH of three continuous examinations was excellent with a coefficient of variation of 3.4 ± 2.0 and an intraclass correlation coefficient of 0.95 [9]. Aizawa et al. also reported that the MBR of the ONH tissue was strongly correlated with the visual field sensitivity in eyes with glaucoma [3]. In addition, Maekubo et al. reported that measurements of the ONH circulation by LSFG could be used for differentiating nonarteritic ischemic optic neuropathy from anterior optic neuritis [19]. Based on these findings, measuring the ONH circulation by LSFG can be considered a useful method in the clinic.

Yamada et al. measured the MBR on the ONH in eyes with untreated CRVO [11]. The corrected MBR was calculated to exclude the influence of the systemic circulation and blood flow of the ONH tissue. After obtaining the MBR values by subtracting the MT from the MV, the ONH blood flow was evaluated by taking the MBR values of the affected eye divided by the MBR values of the healthy fellow eye. The authors reported that this corrected MBR was significantly correlated with the arteriovenous passage time [11]. Similarly, a significant correlation was found between the corrected MBR and arteriovenous passage time in the present study. The strong correlation between the corrected MBR and arteriovenous passage time, which has been used as an indicator of retinal circulation, confirms that the method we used is a highly reliable way to evaluate the circulatory status of the ONH. In addition, Yamada et al. reported that the corrected MBR values in large ONH vessels were significantly correlated with the aqueous VEGF concentrations in eyes with CRVO [11].

It is sometimes difficult to distinguish the vessel and tissue areas at the ONH in eyes with CRVO, because dilatation or blood stasis of capillary vessel on ONH obscures the boundary between the large vessel and tissue on the LSFG images. Thus, the MV and MT values measured in eyes with CRVO may include a measurement error. However, the corrected MBR in the present study was significantly correlated with not only arteriovenous passage time on FA but also the venous tortuosity index on fundus photograph, which suggests that this measurement error is considered to be small.

Nitta et al. reported that there was no significant change in the MBR measured by LSFG in the retinal artery, retinal vein, or ONH after a single IVB injection in eyes with a BRVO [22]. Nagaoka et al. also reported no significant changes in the retinal blood flow measured by laser Doppler velocimetry after a single IVB injection for BRVO [12]. Consistent with these findings, our results showed no significant differences in the corrected MBR after IVB or IVR in eyes with a CRVO. On the other hand, Matsumoto et al. reported 3 cases of ME secondary to nonischemic CRVO in which the blood flow increased after IVBs [13]. In their case, the MBR increased in 3 or 4 weeks after the IVB was confirmed by LSFG, but long term changes of the MBR and exact relationship between the MBR and visual prognosis were unclear.

This study has several limitations. First, the sample size was small, and the follow-up period was short. Further studies with a larger sample size and longer follow-up periods would be required to confirm our findings. We also need to keep in mind that when using LSFG the MBR is a relative value of the blood flow velocity and not the absolute value. Therefore, when comparing the difference between eyes, we used the corrected MBR value. The other limitation is that the present study included both patients treated with IVB and those with IVR. Lastly, Mayama et al. reported that phenylephrine eye drops can depress the ONH circulation [23]. In our study, Mydrin P, tropicamide and phenylephrine, was used prior to the LSFG testing to obtain more accurate measurements of the ONH circulation with LSFG. Thus, there is a possibility that phenylephrine may have influenced the ONH circulation. However, because Mydrin P was administered to both the affected and unaffected eyes and ONH circulation was evaluated using the equation; the MBR of the affected eye/MBR of the unaffected eye, the magnitude of this influence may be small.

## Conclusions

The blood flow velocity on the ONH was significantly correlated with the BCVA and retinal sensitivity before and after anti-VEGF therapy for CRVO. We suggest that the blood flow velocity of the ONH before the therapy is a predictive factor for the visual outcome after treatment for CRVO.

## Ethics and consent to participate

Approval was obtained from the Institutional Review Board of Saneikai Tsukazaki Hospital prior to beginning this study, and the patients gave their written informed consent prior to their inclusion. The procedures used in this study adhered to the tenets of the Declaration of Helsinki.

## Consent to publish

The patients have provided permission to publish clinical data of their case in this study.

## Additional file

Additional file 1: Raw data (MBR, visual function). (DOC 68 kb)

## Abbreviations

asb: apostilb
BCVA: best-corrected visual acuity
BRVO: branch retinal vein occlusion
CRVO: central retinal vein occlusion
DBP: diastolic blood pressure
FA: fluorescein angiography
IOP: intraocular pressure
IVB: intravitreal bevacizumab
IVR: intravitreal ranibizumab
LSFG: laser speckle flowgraphy
MAIA: macular integrity assessment
MAP: mean arterial pressure
MBR: mean blur rate
ME: macular edema
MOPP: mean ocular perfusion pressure
MT: mean blur rate of tissue area
MV: mean blur rate of vessel area
OCT: optical coherence tomography
ONH: optic nerve head
SBP: systolic blood pressure
SD-OCT: spectral-domain optical coherence tomography
UMIN: University hospital Medical Information Network
VEGF: vascular endothelial growth factor
