# Supplementary material for: Correlation between optic nerve head circulation and visual function before and after anti-VEGF therapy for central retinal vein occlusion: prospective, interventional case series
Source: BMC Ophthalmol. 2016 Apr 5;16:36. doi: 10.1186/s12886-016-0211-7 (PMC4820868; doi:10.1186/s12886-016-0211-7)
Supplement: Additional file 1: — Raw data (MBR, visual function). (DOC 68 kb) [file 12886_2016_211_MOESM1_ESM.doc]

Raw data of corrected MBR, visual acuity, and retinal sensitivity

| C-MBR-Pre | C-MBR-1M | C-MBR-3M | C-MBR-6M | VA-Pre | VA-1M | VA-3M | VA-6M | RS-Pre | RS-1M | RS-3M | RS-6M |
| --- | --- | --- | --- | --- | --- | --- | --- | --- | --- | --- | --- |
| .404306 | .453883 | .442500 | .534146 | .5228787 | .5228787 | 1.0000000 | 1.0000000 | 14.4 | 15.2 | 7.6 | 10.6 |
| .483333 | .575000 | .606498 | .653571 | .3010300 | -.0791812 | -.0791812 | -.0791812 | 22.8 | 24.5 | 25.4 | 26.3 |
| .977876 | .903890 | .942263 | .964200 | .2218488 | -.1760913 | .2218488 | .0000000 | 24.5 | 26.6 | 25.5 | 24.0 |
| .816619 | .750742 | .922388 | .771186 | .8239087 | .1549020 | .0969100 | -.0791812 | 16.6 | 15.7 | 19.4 | 21.3 |
| .555970 | .545788 | .556291 | .658784 | .8239087 | .3979400 | .5228787 | .3010300 | 6.6 | 10.1 | 18.8 | 18.8 |
| .191781 | .359848 | .120755 | .172840 | 1.5228787 | 1.1549020 | .6989700 | .6989700 | .0 | 7.5 | 8.8 | 8.8 |
| .437063 | .385159 | .451852 | .387218 | .3010300 | .3979400 | .3979400 | .3979400 | 18.3 | 19.9 | 22.5 | 22.6 |
| .832031 | .820144 | .767790 | .826715 | .3010300 | .1549020 | .0969100 | .0000000 | 19.1 | 22.8 | 23.9 | 24.5 |
| .760797 | .737342 | .661972 | .452663 | .2218488 | .5228787 | .0969100 | .1549020 | 23.6 | 22.5 | 23.4 | 23.2 |
| .557641 | .372159 | .456522 | .543478 | 2.0000000 | .6989700 | 1.0000000 | .3979400 | 2.5 | 16.7 | 19.5 | 21.5 |
| .412214 | .473418 | .447942 | .441026 | 1.6989700 | 1.3010300 | 1.3010300 | 1.3979400 | .0 | 5.4 | 3.2 | 3.0 |
| .247002 | .348131 | .367171 | .301370 | 1.3010300 | 1.0000000 | .6989700 | .6989700 | 9.3 | 8.3 | 19.5 | 20.8 |
| .619898 | .403423 | .560897 | .409222 | 1.0000000 | .5228787 | .3979400 | .3979400 | 16.9 | 19.3 | 22.9 | 23.5 |
| .789790 | .903896 | .630854 | .736070 | .1549020 | .0000000 | .1549020 | .0000000 | 21.9 | 27.0 | 25.9 | 24.5 |
| .763158 | .886986 | .928814 | .858357 | .0969100 | .0457575 | -.1760913 | -.0791812 | 20.1 | 23.2 | 24.5 | 25.8 |
| .717742 | .335917 | .367188 | .277202 | 1.3010300 | .6989700 | .1549020 | .1549020 | 6.7 | 7.3 | 17.9 | 16.7 |
| .830882 | .668258 | .711628 | .809877 | .6989700 | .1549020 | .0000000 | .0457575 | 26.4 | 26.4 | 27.7 | 25.2 |
| .767045 | .820639 | .970588 | .775510 | .3010300 | .0457575 | .0000000 | .2218488 | 21.8 | 26.6 | 28.5 | 24.1 |
| .584746 | .423729 | .550676 | .672619 | .8239087 | .5228787 | .5228787 | .3979400 | 10.9 | 18.8 | 24.0 | 22.9 |
| .352751 | .383871 | .429487 | .179567 | .8239087 | .8239087 | .8239087 | 1.5228787 | 6.2 | 11.9 | 19.9 | 16.3 |
| .607692 | .737542 | .627986 | .652174 | .6989700 | .0000000 | -.0791812 | -.0791812 | 21.5 | 26.9 | 28.1 | 27.6 |
| .145773 | .120000 | .155620 | .317961 | 1.0000000 | .5228787 | .5228787 | .5228787 | 15.0 | 16.8 | 17.8 | 17.8 |
| .608527 | .497696 | .656000 | .605505 | .6989700 | .5228787 | .3979400 | .2218488 | 14.9 | 8.7 | 12.3 | 13.0 |
| .735043 | .822034 | 1.022727 | .684932 | .3979400 | .3010300 | .3010300 | .3979400 | 15.3 | 23.0 | 26.5 | 26.5 |
| .936441 | .846809 | .612903 | .990244 | .2218488 | .0000000 | .0000000 | .0000000 | 12.6 | 17.6 | 21.1 | 16.7 |
| .588060 | .362857 | .345609 | .347339 | .3979400 | .3979400 | .3010300 | .0000000 | 19.7 | 21.6 | 19.4 | 24.2 |
| .863850 | .567568 | .552204 | .607306 | .1549020 | .0457575 | .3010300 | .2218488 | 24.3 | 27.4 | 26.6 | 22.4 |
| .250000 | .560081 | .523232 | .502913 | 1.0457575 | .6989700 | .6989700 | 1.0000000 | 11.6 | 20.9 | 22.6 | 20.1 |
| 1.364662 | .992509 | 1.046763 | 1.456311 | .3010300 | .2218488 | .3010300 | .0457575 | 19.8 | 22.2 | 23.5 | 23.9 |
| .443548 | .447876 | .496032 | .530364 | .3979400 | .3010300 | .3010300 | .3010300 | 5.2 | 16.2 | 17.4 | 8.8 |
| .947791 | .970954 | .870293 | 1.240157 | .3979400 | .1549020 | .0969100 | .0000000 | 23.2 | 24.1 | 23.5 | 26.0 |

C-MBR, Corrected MBR; VA, Visual acuity; RS, Retinal sensitivity.
